# Seroprevalence of SARS-CoV-2 in a Large Cohort of Italian Police Officers

**DOI:** 10.3390/ijerph182212201

**Published:** 2021-11-20

**Authors:** Sergio Garbarino, Alexander Domnich, Elisabetta Costa, Irene Giberti, Stefano Mosca, Cristiano Belfiore, Fabrizio Ciprani, Giancarlo Icardi

**Affiliations:** 1 Italy State Police Health Service Department, Ministry of Interior, 00198 Rome, Italy; cristiano.belfiore@poliziadistato.it (C.B.); fabrizio.ciprani@interno.it (F.C.); 2 Post-Graduate School of Occupational Medicine, Università Cattolica del Sacro Cuore, 00168 Rome, Italy; 3 Hygiene Unit, San Martino Policlinico Hospital-IRCCS for Oncology and Neurosciences, 16132 Genoa, Italy; alexander.domnich@hsanmartino.it (A.D.); icardi@unige.it (G.I.); 4 Department of Health Sciences (DISSAL), University of Genoa, 16132 Genoa, Italy; ec240583@gmail.com (E.C.); irene.giberti25@gmail.com (I.G.); stefanom@unige.it (S.M.)

**Keywords:** COVID-19, SARS-CoV-2, seroprevalence, police officers, occupational exposure, Italy

## Abstract

Certain professional categories are at a high occupational exposure to COVID-19. The aim of this survey was to quantify the seroprevalence of SARS-CoV-2 among police officers in Italy and identify its correlates. In this cross-sectional study, a nationally representative sample of State police employees was tested for IgG and IgM before the start of the National vaccination campaign. A total of 10,535 subjects (approximately 10% of the total workforce) participated in the study. The overall seroprevalence was 4.8% (95% CI: 4.4–5.3%). However, seropositivity was unevenly distributed across the country with a clear (*p* < 0.001) North–South gradient. In particular, the seroprevalence was 5.6 times higher in northern regions than in southern regions (9.0% vs. 1.6%). Most (71.2%) seropositive subjects reported having no recent symptoms potentially attributable to SARS-CoV-2 infection. Previous dysosmia, dysgeusia, and influenza-like illness symptoms were positive predictors of being seropositive. However, the prognostic value of dysosmia depended (*p* < 0.05) on both sex and prior influenza-like illness. The baseline seroprevalence of SARS-CoV-2 in police employees is considerable. A significant risk of occupational exposure, frequent asymptomatic cases and the progressive waning of neutralizing antibodies suggest that the police workers should be considered among the job categories prioritized for the booster COVID-19 vaccine dose.

## 1. Introduction

The ongoing COVID-19 pandemic has led to a devasting public health and socioeconomic crisis worldwide. At the global level, the attack rate and burden of disease indicators are unevenly distributed among different population strata and depend on a variety of characteristics including age, geographic area, risk of exposure, vaccination coverage, etc. [1,2,3,4]. The officially reported burden indicators are, however, significantly underestimated since a high number of cases are not detected and are underreported due to limited testing capacities [5].

The issue of disease underreporting may be at least partially addressed through well-designed seroepidemiological surveys that are able to quantify the susceptible population fraction and can therefore inform disease modelling, forecasting, and optimize vaccination and other public health measures [6]. Indeed, a recent systematic review and meta-analysis [1] has estimated that the true prevalence of SARS-CoV-2 infection may be up to 11 times higher than the officially reported statistics.

Occupational exposure to SARS-CoV-2, especially among first responders and workers in the public sector of primary community interest (e.g., healthcare, transport and police), is of concern. The prevalence of anti-SARS-CoV-2 antibodies among healthcare workers (HCWs) has received particular attention [3]. Data on other professional categories are limited and usually restricted to small local studies that demonstrated a great variability of seroprevalence between workers of different sectors [7,8]. For instance, in northern Italy the highest seroprevalence before immunization was among workers of long-term care facilities, logistics and some types of factories [8].

In Italy, the police force is considered a target group for some free-of-charge vaccinations, including seasonal influenza [9] and the schedule of COVID-19 vaccines [10]. Indeed, as part of their crucial community role, most State police officers (SPOs) have frequent close contacts with the general public and continue working during lockdowns. However, nationally representative seroepidemiological surveys have been conducted for this professional category as of yet. A study [11] carried out in the metropolitan area of Milan (Lombardy, northern Italy) between May and October 2020 reported an IgM and/or IgG seroprevalence in police workers of 5.5%, which was found to be higher than that of office workers (3.6%), but lower than that of HCWs (12.2%). A small study by De Santi et al. [12] reported that police officers in the region of Marche (central Italy) showed a significant increase in the likelihood of testing positive for SARS-CoV-2, as compared with some other professional categories.

The objective of this study was to quantify the seroprevalence of SARS-CoV-2 in a representative and large sample of SPOs in Italy, and to identify some correlates of seropositivity, which would be useful for establishing future preventive measures, including an eventual booster COVID-19 vaccine dose.

## 2. Materials and Methods

### 2.1. Overall Study Design, Setting and Participants

This cross-sectional study was conducted between April and July 2020, i.e., immediately after the peak (10 March 2020) of the first pandemic wave [13] and before the start of the National vaccination campaign in late December 2020. According to GISAID (www.gisaid.org (accessed on 17 November 2021)), during the study period in Italy most (91%) SARS-CoV-2 isolates led to a D614G mutation in the (S)pike protein.

The study population was composed of approximately 100,000 units of SPOs. The inclusion criteria were as follows: subjects had to be on duty, not previously tested for anti-SARS-CoV-2 antibodies and provide an informed consent. No exclusion criteria were applied.

In order to obtain a geographically representative sample, subjects were enrolled in three macro-areas, namely northern (regions of Lombardy, Veneto and Emilia Romagna), central (regions of Tuscany, Latium and Marche) and southern (regions of Campania and Apulia) Italy.

By assuming a true seroprevalence of 5% (precision of 1%) with a conservative test sensitivity and specificity of 75% and 90%, respectively, we aimed to enroll at least 10,451 participants (i.e., approximately 10% of the total workforce).

Participation in this study was voluntary and anonymity was guaranteed. The study was conducted according to the guidelines of the Declaration of Helsinki. The survey was approved by the competent Ethics Committee (Prot. #0036646).

### 2.2. Study Procedures and Outcome

Following a physical examination, venous blood samples were drawn from participants. One sample per was collected subject. Seroprevalence was evaluated with a commercially available qualitative lateral flow assay COVID-19 IgG/IgM Rapid Test Cassette (Zhejiang Orient Gene Biotech Co Ltd., Huzhou, Zhejiang, China) according to the manufacturer’s instructions. The assay detects anti-S1 and other responses. To summarize, 10 μL of whole blood was applied and results were read after 10–15 min. The manufacturer’s declared sensitivity for IgG and IgM are 97.2% and 87.9%, respectively, while the specificity is 100% for both. However, in a recent meta-analysis [14] the sensitivity for the IgG and the IgM of this kit was found to be lower. Subjects with detectable IgG and/or IgM were deemed seropositive. However, considering that the sensitivity of lateral flow immunoassays for IgM is substantially lower than for IgG and IgG/IgM [15], we conducted a sensitivity analysis by including only subjects with a detectable IgG response.

During the visit, participants were also interviewed about the presence of any underlying morbidities, the recent (from January 2020) presence of olfactory and gustatory dysfunctions, or influenza-like illness (ILI) symptoms. ILI was defined according to the World Health Organization (WHO) definition [16] as “an acute respiratory illness with a measured temperature of ≥38 °C and cough”. The risk of occupational exposure was measured against working patterns, i.e., predominantly office-based or field-based.

### 2.3. Data Analysis

Seroprevalence was expressed as percentage with 95% confidence intervals (CIs). The difference in seropositivity status according to the independent variables considered (age, sex, geographic area, frequent contacts with public, presence of chronic conditions, olfactory and gustatory dysfunctions, and ILI symptoms) was compared by means of the chi-squared test and the effect size was expressed as odds ratio (OR). Multivariable logistic regression was then applied in order to predict the seropositivity status.

Data were analyzed in R stat packages, version 4.0.3 (R Foundation for Statistical Computing, Vienna, Austria) [17].

## 3. Results

A total of 10,535 subjects underwent serological testing and their principal characteristics are reported in Table 1. In summary, most participants were males, healthy, predominantly field-based and their mean age was 45.4 years (range 19–66 years). A recent ILI was reported by 4.5% of police employees, while symptoms of dysosmia or dysgeusia were reported by around 1% of the participants (Table 1).

**Figure 1:**
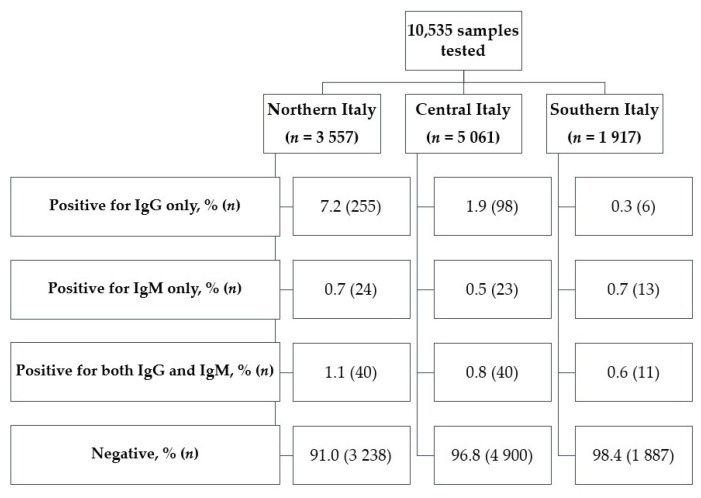
Distribution of seropositive and seronegative police officers, by macro-region.

The overall seroprevalence was 4.8% (95% CI: 4.4–5.3%). Among seropositive subjects (*n* = 510), the IgG response was 3 times higher than the IgM response [88.6% (95% CI: 85.5–91.3%) vs. 29.6% (95% CI: 25.7–33.8%)]. As shown in Table 2, there was a clear North-South gradient, as the seroprevalence was 9.0% (95% CI: 8.0–10.0%), 3.2% (95% CI: 2.7–3.7%) and 1.6% (95% CI: 1.1–2.2%) in northern, central and southern regions, respectively (*p* < 0.001). Moreover, SPOs residing in southern regions showed comparatively high IgM reactivity, suggesting a later spread of SARS-CoV-2 in Southern Italy (Figure 1). Subjects reporting recent ILI symptoms, olfactory and gustatory dysfunctions showed significantly increased odds of being seropositive to IgG and/or IgM (Table 2). Among seropositive participants, 71.2% (95% CI: 67.0–75.1%) were completely asymptomatic in the period from the start of pandemic to the blood test.

In the multivariable logistic model (Table 3), the main effect of the independent variables of geographic area, prior olfactory and gustatory dysfunctions and ILI were significantly associated with a positive test result. Moreover, two interaction terms proved statistically significant. First, males with dysosmia had a 91.9% probability of testing seropositive [adjusted OR (aOR) = 11.35], while the probability of seropositivity in females was 97.3% (aOR = 35.60). Secondly, olfactory dysfunction in patients with no ILI history had a higher prognostic value (aOR = 11.35) compared with those who reported some ILI symptoms (aOR = 4.35).

Finally, in the sensitivity analysis which included only subjects with IgG antibodies (*n* = 452), no major changes occurred (Table 4). Furthermore, as shown by the Akaike information criterion (AIC), the model fit improved substantially (AICs of 3521 and 3144 for the base-case and sensitivity analysis models, respectively).

## 4. Discussion

To our knowledge, this study is among the first to quantify the baseline risk of exposure to SARS-CoV-2 in a large, nationally representative cohort of SPOs. These findings may be useful for planning effective preventive strategies (e.g., administration of the booster COVID-19 vaccine dose) for this first responder occupational category.

Our results showed that the risk of exposure to SARS-CoV-2 in SPOs is higher than in the general population, and is close to that of HCWs; this latter group is a well-recognized occupational risk category [3,10,11]. As of April 2020, seroprevalence estimates during the first pandemic wave among HCWs in Lombardy ranged from 5.1% [18] to 9.4% (95% CI: 3.9–10.6%) [19]. In the present survey, while the overall seroprevalence was 4.8%, it reached 9.0% in subjects working in the northern Italian regions. We then observed a clear North–South gradient with southern regions displaying significantly lower seroprevalence (1.6%), which resembles the distribution of cases during the first pandemic wave. Again, compared with the proportion observed in our study, previous reports from southern Italy [20,21] showed a lower seroprevalence in the general population, but a similar estimate among HCWs. For example, in Apulia the seroprevalence rate was 0.99% among blood donors (May 2020) [20] and 1.9% in HCWs (March–May 2020) [21]. Analogously, a nationwide seroprevalence study commissioned by the Italian Ministry of Health and National Institute of Statistics and conducted across a similar time period (25 May–15 July 2020), revealed the highest seroprevalence in Lombardy (7.5%) which ranked first, while estimates in all southern regions were <1% [22]. The most plausible reason for the observed North–South gradient is a progressive SARS-CoV-2 diffusion southward from the initial disease outbreak in Lombardy [7]. This is further corroborated by a relatively high IgM reactivity among SPOs residing in the southern regions. Some environmental factors (e.g., southern regions have higher average temperature regimens) may have also contributed to this gradient [23]. 

According to a recent Circular issued by the Italian Ministry of Health [24], only the professional category of HCWs will be offered a booster COVID-19 vaccine dose. Our data indicate that SPOs have a significant risk of exposure and should therefore be prioritized for the booster dose. Indeed, available studies [25,26] suggest a significant waning of anti-S and anti-spike IgG and neutralizing antibodies following the primary 2-dose schedule of both the BNT162b2 and ChAdOx1 vaccines. Similarly, following natural infection, the anti-S antibody response declines over time [27]. Notably, it has been demonstrated [26] that neutralizing antibody titers decline more quickly among men than among women [ratio of means 0.64 (95% CI: 0.55–0.75)]; male SPOs represented about four fifth of the total workforce. 

About three quarters (71.2%) of seropositive SPOs were fully asymptomatic. A similar proportion of asymptomatic cases was observed among Italian [11], American [28] and Brazilian [29] police officers. As shown in a systematic review by Sah et al. [30], the asymptomaticity rate may range from 4% to 90% depending on symptoms, age (higher age is associated with lower asymptomaticity), and background health status (subjects with comorbidities have lower asymptomaticity). A relatively high proportion of asymptomatic individuals among SPOs may therefore be explained by the fact that these workers are typically young adults and generally healthier than the general population of the same age [31]. Indeed, in our survey, only 5.6% of participants had at least one morbidity, while in the Italian population this proportion is 19.2% among 20–24-year-olds and reaches 60.9% among adults aged 60–64 years [32].

As expected, the presence of recent olfactory and gustatory dysfunctions and ILI were directly associated with positive serology. A previous study conducted among Italian adults [33] reported significantly higher odds of seropositivity in subjects with prior ILI and anosmia and/or ageusia, while the demographic variables of age and sex were not found to be statistically significant in the adjusted model. On the other hand, we established that dysosmia alone has a higher predictive value than dysosmia with ILI symptoms. SARS-CoV-2 infected patients may present a new sudden onset of dysosmia without any other symptom [34] and may act as a warning sign of early-stage COVID-19 [35]. This finding may therefore be useful for the clinical differential diagnosis, especially in low-resource settings. Finally, the association between olfactory dysfunction and positive serology was significantly higher in female workers. In this regard, the available systematic evidence [36] suggests that the prevalence of anosmia is higher in women.

Despite the representativeness of the sample and a large sample size, this study may suffer from some limitations. First, under the lower test sensitivity assumption, true seroprevalence may be higher [37]. Indeed, the sensitivity of lateral flow immunoassays for IgG is still suboptimal [15], especially during the first two weeks following exposure. Secondly, the dichotomization rule of the working pattern (office-based vs. field-based) applied may be reductionist and prone to the subjectivity of judgement. Another explanation could lie in frequent between-colleague contact, i.e., when the field-based SPOs may infect their office-based counterparts during, for example, internal meetings. Therefore, the observed non-difference may be biased. On the other hand, a recent study conducted among Polish police officers [38] has similarly did not find a significant difference in seropositivity to SARS-CoV-2 between office-based and fieldwork employment patterns. Third, the recall bias could not be ruled out, especially concerning the reporting of prior ILI symptoms. Fourth, for some organizational and logistical issues, it was not possible to reach the desired sample size in a shorter time period. This means that seroprevalence estimates in April 2020 are likely to be lower than in July 2020. 

In conclusion, the present survey quantified the pre-vaccination prevalence of antibodies to SARS-CoV-2 in a large representative cohort of Italian police workers. The seroprevalence was relatively high, which suggests that exposure in this occupational category is considerable. The relatively high rate of asymptomatic cases makes it difficult to apply contact tracing and other containment strategies successfully. Indeed, the etiological diagnosis of SARS-CoV-2 in this occupational category is likely underperformed; seroepidemiological studies are therefore useful for obtaining the susceptible population fraction. Considering both the relatively high occupational exposure and progressive waning of the neutralizing antibodies, the booster COVID-19 vaccine dose should also be prioritized for police employees.

## Figures and Tables

**Table 1:** Characteristics of the study participants (*n* = 10,535).

Characteristic	Level	% (*n*)	95% CI
Age, years ^1^	Mean	45.4	9.5
Sex	Male	81.3 (8562)	80.5–82.0
	Female	18.7 (1973)	18.0–19.5
Geographic area	North	33.8 (3557)	32.9–34.7
	Center	49.0 (5161)	48.0–49.9
	South	18.2 (1917)	17.5–18.9
Working pattern	Predominantly field-based	80.5 (8483)	79.8–81.3
	Predominantly office-based	19.5 (2052)	18.7–20.2
Chronic conditions	Yes	5.6 (588)	5.2–6.0
	No	94.4 (9947)	94.0–94.8
Recent ILI	Yes	4.5 (476)	4.1–4.9
	No	95.5 (10,059)	95.1–95.9
Recent olfactory dysfunction	Yes	1.2 (122)	1.0–1.4
	No	98.8 (10,413)	98.6–99.0
Recent gustatory dysfunction	Yes	0.9 (94)	0.7–1.1
	No	99.1 (10,441)	98.9–99.3

^1^ Results are reported as mean and standard deviation (standard deviation). ILI, influenza-like illness.

**Table 2:** Comparison between seropositive and seronegative subjects.

Characteristic	Level	IgG and/or IgM status, % (95% CI)		OR (95% CI)
		Positive (*n* = 510)	Negative (*n* = 10,025)	
Age, years ^1^	Mean	45.5 (9.7)	45.4 (9.5)	1.00 (0.99–1.01) ^2^
Sex	Male	76.5 (72.5–80.1)	81.5 (80.7–82.3)	Ref
	Female	23.5 (19.9–27.5)	18.5 (17.7–19.3)	1.36 (1.10–1.68) **
Geographic area	South	5.9 (4.0–8.3)	18.8 (18.1–19.6)	Ref
	Center	31.6 (27.6–35.8)	48.9 (47.9–49.9)	2.07 (1.39–3.06) ***
	North	62.5 (58.2–66.8)	32.3 (31.4–33.2)	6.20 (4.24–9.05) ***
Working pattern	Predominantly office-based	22.9 (19.4–26.8)	19.3 (18.5–20.1)	Ref
	Predominantly field-based	77.1 (73.2–80.6)	80.7 (79.9–81.5)	0.80 (0.65–0.99) *
Chronic conditions	No	94.5 (92.2–96.3)	94.4 (93.9–94.9)	Ref
	Yes	5.5 (3.7–7.8)	5.6 (5.2–6.1)	0.98 (0.66–1.45)
Recent ILI	No	77.3 (73.4–80.8)	96.4 (96.0–96.8)	Ref
	Yes	22.7 (19.2–26.6)	3.6 (3.2–4.0)	7.90 (6.27–9.97) ***
Recent olfactory dysfunction	No	85.9 (82.6–88.8)	99.5 (99.3–99.6)	Ref
	Yes	14.1 (11.2–17.4)	0.5 (0.4–0.7)	32.79 (22.58–47.63) ***
Recent gustatory dysfunction	No	89.8 (86.8–92.3)	99.6 (99.4–99.7)	Ref
	Yes	10.2 (7.7–13.2)	0.4 (0.3–0.6)	26.99 (17.78–40.96) ***

^1^ Results are reported as mean and standard deviation (SD); ^2^ 1-year increase; *** *p* < 0.001; ** *p* < 0.01; * *p* < 0.05. ILI, influenza-like illness.

**Table 3:** Multivariable logistic regression model to predict the SARS-CoV-2 seropositivity status (*n* = 510).

Characteristic	Level	*b* (SE)	aOR (95% CI)	*p*
Intercept	–	−4.614 (0.338)	0.01 (0.01–0.02)	<0.001
Age	1-year increase	0.009 (0.005)	1.01 (1.00–1.02)	0.10
Sex	Male	Ref	Ref	Ref
	Female	0.063 (0.125)	1.07 (0.83–1.36)	0.62
Geographic area	South	Ref	Ref	Ref
	Center	0.453 (0.206)	1.57 (1.05–2.35)	0.028
	North	1.651 (0.197)	5.21 (3.54–7.67)	<0.001
Working pattern	Predominantly office-based	Ref	Ref	Ref
	Predominantly field-based	−0.043 (0.119)	0.96 (0.76–1.21)	0.72
Chronic conditions	No	Ref	Ref	Ref
	Yes	0.138 (0.212)	1.15 (0.76–1.74)	0.51
Recent influenza-like illness	No	Ref	Ref	Ref
	Yes	1.623 (0.148)	5.07 (3.79–6.77)	<0.001
Recent olfactory dysfunction	No	Ref	Ref	Ref
	Yes	2.451 (0.381)	11.60 (5.49–24.51)	<0.001
Recent gustatory dysfunction	No	Ref	Ref	Ref
	Yes	0.792 (0.324)	2.21 (1.17–4.17)	0.015
Sex × olfactory dysfunction	–	1.102 (0.475)	3.01 (1.19–7.64)	0.020
ILI × olfactory dysfunction	–	−0.988 (0.434)	0.37 (0.16–0.87)	0.023

ILI, influenza-like illness.

**Table 4:** Sensitivity analysis including only subjects with the detectable IgG response (*n* = 452).

Characteristic	Level	*b* (SE)	aOR (95% CI)	*p*
Intercept	–	−4.982 (0.379)	0.01 (0.00–0.01)	<0.001
Age	1-year increase	0.007 (0.006)	1.01 (1.00–1.02)	0.22
Sex	Male	Ref	Ref	Ref
	Female	−0.077 (0.139)	0.93 (0.71–1.21)	0.58
Geographic area	South	Ref	Ref	Ref
	Center	0.732 (0.252)	2.08 (1.27–3.41)	0.004
	North	2.018 (0.243)	7.53 (4.68–12.11)	<0.001
Working pattern	Predominantly office-based	Ref	Ref	Ref
	Predominantly field-based	−0.050 (0.126)	0.95 (0.74–1.22)	0.69
Chronic conditions	No	Ref	Ref	Ref
	Yes	0.186 (0.225)	1.20 (0.77–1.87)	0.41
Recent influenza-like illness	No	Ref	Ref	Ref
	Yes	1.702 (0.153)	5.49 (4.07–7.40)	<0.001
Recent olfactory dysfunction	No	Ref	Ref	Ref
	Yes	2.356 (0.394)	10.54 (4.87–22.82)	<0.001
Recent gustatory dysfunction	No	Ref	Ref	Ref
	Yes	0.975 (0.327)	2.65 (1.40–5.03)	0.003
Sex × olfactory dysfunction	–	1.414 (0.487)	3.14 (1.21–8.16)	0.004
ILI × olfactory dysfunction	–	−1.062 (0.442)	0.35 (0.15–0.82)	0.016

ILI, influenza-like illness.

## Data Availability

Data used in this study may be obtained from the corresponding author upon a reasonable request and prior permission of the Italian Ministry of Interior.
